# A comprehensive survey of human polymorphisms at conserved splice dinucleotides and its evolutionary relationship with alternative splicing

**DOI:** 10.1186/1471-2148-10-122

**Published:** 2010-04-30

**Authors:** Makoto K Shimada, Yosuke Hayakawa, Jun-ichi Takeda, Takashi Gojobori, Tadashi Imanishi

**Affiliations:** 1 Biomedicinal Information Research Center, National Institute of Advanced Industrial Science and Technology, 2-42 Aomi Koto-ku, Tokyo135-0064, Japan; 2 Japan Biological Informatics Consortium, 10F TIME24 Building, 2-45 Aomi, Koto-ku, Tokyo 135-0064, Japan; 3 Institute for Comprehensive Medical Science, Fujita Health University, 1-98 Dengakugakubo, Kutsukake-cho, Toyoake, Aichi 470-1192, Japan; 4 Hitachi Software Engineering Co., Ltd., 1-1-43 Suehirocho, Tsurumi-ku, Yokohama 230-0045, Japan; 5 Center for Information Biology and DNA Data Bank of Japan, National Institute of Genetics, 1111 Yata, Mishima, Shizuoka 411-8540, Japan

## Abstract

**Background:**

Alternative splicing (AS) is a key molecular process that endows biological functions with diversity and complexity. Generally, functional redundancy leads to the generation of new functions through relaxation of selective pressure in evolution, as exemplified by duplicated genes. It is also known that alternatively spliced exons (ASEs) are subject to relaxed selective pressure. Within consensus sequences at the splice junctions, the most conserved sites are dinucleotides at both ends of introns (splice dinucleotides). However, a small number of single nucleotide polymorphisms (SNPs) occur at splice dinucleotides. An intriguing question relating to the evolution of AS diversity is whether mutations at splice dinucleotides are maintained as polymorphisms and produce diversity in splice patterns within the human population. We therefore surveyed validated SNPs in the database dbSNP located at splice dinucleotides of all human genes that are defined by the H-Invitational Database.

**Results:**

We found 212 validated SNPs at splice dinucleotides (sdSNPs); these were confirmed to be consistent with the GT-AG rule at either allele. Moreover, 53 of them were observed to neighbor ASEs (AE dinucleotides). No significant differences were observed between sdSNPs at AE dinucleotides and those at constitutive exons (CE dinucleotides) in SNP properties including average heterozygosity, SNP density, ratio of predicted alleles consistent with the GT-AG rule, and scores of splice sites formed with the predicted allele. We also found that the proportion of non-conserved exons was higher for exons with sdSNPs than for other exons.

**Conclusions:**

sdSNPs are found at CE dinucleotides in addition to those at AE dinucleotides, suggesting two possibilities. First, sdSNPs at CE dinucleotides may be robust against sdSNPs because of unknown mechanisms. Second, similar to sdSNPs at AE dinucleotides, those at CE dinucleotides cause differences in AS patterns because of the arbitrariness in the classification of exons into alternative and constitutive type that varies according to the dataset. Taking into account the absence of differences in sdSNP properties between those at AE and CE dinucleotides, the increased proportion of non-conserved exons found in exons flanked by sdSNPs suggests the hypothesis that sdSNPs are maintained at the splice dinucleotides of newly generated exons at which negative selection pressure is relaxed.

## Background

Pre-mRNA splicing of eukaryotes requires three basic signals (splicing motifs) for the recognition of introns. The splicing motifs are the 5' intron end (donor) and the 3' intron end (acceptor), and the branch site. The splicing motifs at the 5' and 3' splice sites, known as "ag|GTragt" ("|" is the splice junction; "r" is a or g) and "(y)_12-17_nAG|g" ("y" is c or t; "n" is a, t, g or c; and subscript indicates the repeat number) [1,2]. A human expressed sequence tag-based study showed that 99.24% and 0.69% of introns are flanked by GT-AG and GC-AG dinucleotides (splice dinucleotides), respectively [3]. Other types of splice dinucleotides are also found in the human genome; these are AT-AC(0.05%) and others (0.02%) [3]. Irrespectively of these variations at the splice dinucleotides, there are two well-studied splicing mechanisms [4]. One mechanism utilizes a major spliceosome, an assembly of five small nuclear ribonucleoprotein particles (U1, U2, U4, U5, and U6 snRNP). The other mechanism uses the minor spliceosome, consisting of U11, U12, U4atac, U5, and U6atac, instead. Thus, most of exons are flanked by the virtually "invariant" GT and AG dinucleotides (splice dinucleotides) [1]. Other additional splicing motifs, such as enhancers and silencers located in exons and introns have vast variety in motif signals and locations, but contribute to splicing fidelity.

Some single base-pair substitutions occurring at the "invariant" splice dinucleotides cause alteration of splice patterns and are associated with serious diseases, for example, NF1 [5], GSTM4 [6], cyclin D1 [7], NUDT1(MTH1) [8], and LDLR [9], (for review, see [10,11]). The Human Genome Mutation Database (HGMD) at the Institute of Medical Genetics in Cardiff [12,13] has annotated a total of 9267 entries for mutations in the vicinity of splice sites, which include 2362, 756, 1199, and 1355 entries for mutations at splice dinucleotides at sites '+1(G)', '+2(T)', '-2(A)', and '-1(G)', respectively [14]. The databases DBASS5 [15] and DBASS3 [16] contain 431 and 283 details of aberrant splice sites, respectively [14], which are generated as a result of disease-causing mutations in humans.

While other single base-pair substitutions at splicing dinucleotides are known to be maintained as single nucleotide polymorphisms (SNPs) in human populations [17,18], the question is: "what determines whether a single base-pair substitution at a splice dinucleotide will be maintained as a SNP or eliminated from the population?"

To address this question, we evaluated SNPs at splice dinucleotides (sdSNPs) in the context of selective pressure in the course of evolution. Generally, functional constraints on exons differ between alternatively spliced exons (ASEs) and constitutively spliced exons (CSEs). ASEs are subject to relaxed negative selective pressure, which is suggested by their significantly higher Ka/Ks values compared with other exons [19]. This relaxation is the most fundamental conceptual constituent of exon creation via alternative splicing (AS) and was first proposed at the time of the discovery of the exon/intron structure in the 1970s [20]. Recent studies revealed that AS is an important mechanism for creating new exons [21-24] and that accelerated accumulation of SNPs at additional splicing motifs after gene duplication enhances exon generation [25].

Current advances in genome informatics and comparative genomics demonstrate that ASEs can be sub-divided into two contrasting categories. When ASEs are classified as conserved or non-conserved in exon structure, low synonymous rates are characteristic of conserved ASEs but not of those with non-conserved exonic structure [26]. Moreover, when they are classified as boundary-shifting (complex) ASEs or non-boundary shifting (simple) ASEs (those of the former type change the exon/intron boundaries of the flanking exons whereas those of the latter type do not), complex ASEs are under stronger selection pressure at the amino acid level but less pressure at the RNA level than CSEs, while reverse trends were observed in simple ASEs [27,28]. These opposite evolutionary effects between different AS patterns have been discussed as a key role of AS in the 'switch-like' regulation of gene expression [29].

If it is supposed that sdSNPs are related to variation within populations in the regulation of gene expression through alterations in splicing patterns, the evolutionary profiles of sdSNP may differ between sdSNPs flanking ASEs (sdSNPs at AE dinucleotides) and those flanking CSEs (sdSNPs at CE dinucleotides).

Here, we extracted the sdSNPs from all human genes, and evaluated them by comparing flanking exon properties between ASEs or CSEs. Each group was subsequently divided into three subgroups according to exon conservation status between human and mouse, namely transcript-conserved, genome-conserved, and non-conserved (See Methods for details of the criteria used). We found that sdSNPs exist in the human genome with high allele frequencies, and with no significant difference in flanking exon properties between ASEs and CSEs. Moreover, we also found that these sdSNPs are prone to be maintained at the splice dinucleotides of newly generated exons. These results suggest that sdSNPs are associated with relaxed selection pressure for newly generated exons.

## Results

### Validated SNPs at splice sites

**Figure 1:**
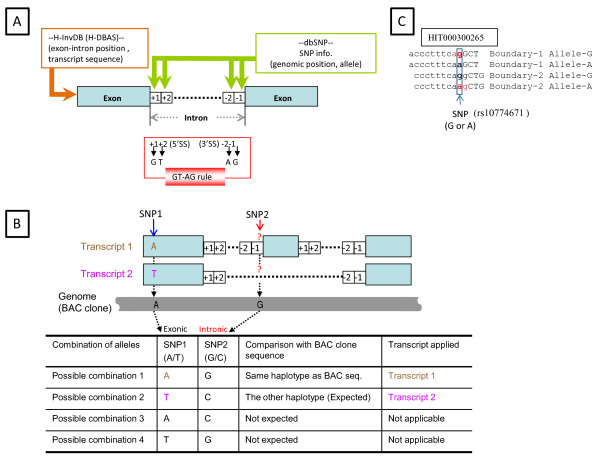
**Method**. (A) Investigated sites with exon/intron boundaries. We searched dbSNP build 127 (green box) for SNPs on splice dinucleotides (+1, +2, -1, -2) at both edges of whole introns of the human genes defined by H-InvDB (orange box). We named these SNPs "sdSNPs", and examined whether their alleles satisfied the GT-AG rule (red box), which requires that the 5' and 3' ends of introns should be GT and AG sequences, respectively. Blue boxes and dotted lines are exonic and intronic regions. (B) Allele estimation method for SNPs at splice dinucleotides. To estimate the SNP (SNP2, red arrow) allele (red question mark) of each transcript sequence that had been spliced out, we used different SNPs (SNP1, blue arrow) located in the nearest exon of the same transcript. Assuming linkage disequilibrium between a SNP at a splice dinucleotide (e.g., SNP2, whose alleles are G and C) and that in an exon (SNP1 whose alleles are A and T), we estimated alleles of SNP2 in transcript 1 (brown) and transcript 2 (pink) using a BAC clone sequence (gray bar). In this case, transcript 1 was estimated as the same haplotype as the BAC sequence because transcript 1 has A at the SNP1 as in BAC sequence. See text (Methods) for detail. (C) Example in which a SNP gives another exon boundary This example shows that the SNP (bold in box) whose alleles are A and G make two possible exon/intron boundaries consistent with the GT-AG rule (Boundary-1 Allele-G and Boundary-2 Allele-A). When the G allele is given, the exon/intron boundary is the next base of the SNP. When A alleles are given, the boundary is shifted one base downstream. The lower-case and capital letters indicate intronic and exonic regions, respectively. The red "ag" indicates a match to 3' splice site sequences.

We surveyed 213 441 transcripts, defined by the H-Invitational Database (H-invDB) 4.6 [30,31], containing 203 673 ASEs and 550 281 CSEs (a total of 753 954 exons) classified on the basis of information on exon usage (i.e., alternative or constitutive) determined by H-DBAS [32-34] (see the Methods section and Figure 1). The numbers of splice sites counted after mapping these transcripts on the genome were 88 308 (176 616 bp), 216 835 (433 670 bp), and 305 143 (610 286 bp) in ASEs, CSEs, and total exons, respectively.

**Figure 2:**
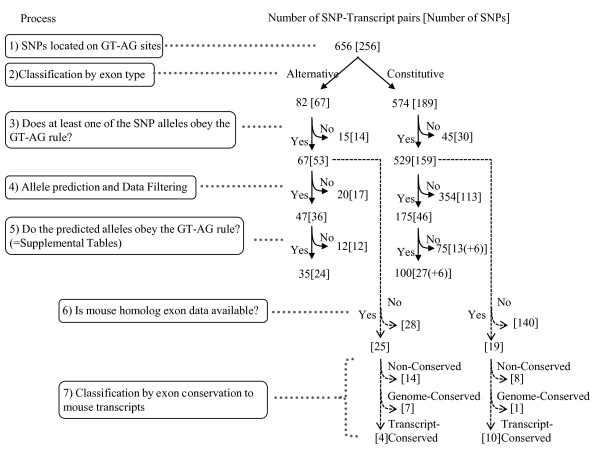
**Data processing flow and results of each step (i.e., numbers of SNP-transcript pairs with number of SNPs included)**. Numbers without brackets are numbers of SNP-transcript pairs that count the number of transcripts including an exon flanking a sdSNP if multiple transcripts are mapped over the SNP position. Brackets indicate the number of SNPs, i.e., the numbers counted uniquely based on the genome position. Parentheses in brackets (Process 5) indicate the number of sdSNPs counted in the categories of consistent and inconsistent with the GT-AG rule. This is because both alleles of these sdSNPs were included in both categories (i.e., consistent and inconsistent with the GT-AG rule) of transcript. All data in the figure are listed in Additional file 1, Table S1.

Figure 2 shows the numbers of transcripts in each filtering process. Numbers in parentheses are the numbers of SNPs or numbers counted uniquely on the genome sequence. Among the total transcripts, we found 596 combinations of transcript and sdSNP in which at least one of the SNP alleles followed the GT-AG rule. Of these, 67 were at AE dinucleotides and 529 were at CE dinucleotides (Additional file 1, Table S1). These combinations of transcript and sdSNP consisted of 53 and 159 for sdSNPs at AE and CE dinucleotides, respectively (Process 3 in Figure 2). We could predict SNP alleles in 47 and 175 transcripts of AE and CE dinucleotides, respectively (Process 4 in Figure 2) and provide information such as gene names, SNP positions, and SNP heterozygosities (Additional file 1, Table S2). Updated data on these sdSNPs will be continuously provided in the VarySysDB database [35,36].

### SNP distribution and density at splice dinucleotides

We calculated the proportions of SNP sites among the total sites at splice dinucleotides. These were 0.030% (53 SNPs/176 616 bp), 0.037% (159 SNPs/433 670 bp), and 0.139% (1 604 284 SNPs/1 150 855 755 bp) for AE dinucleotides, CE dinucleotides, and intron regions, respectively. The densities of sdSNPs in both AE and CE dinucleotides were significantly lower than that in introns, as is generally known (*P *< 0.001 for both of AE dinucleotides vs. introns and CE dinucleotides vs. introns). On the other hand, there was no significant difference between SNP density for sdSNPs at AE and CE dinucleotides (Table 1).

**Table 1:** Comparison of sdSNP properties between ASEs and CSEs

Properties of sdSNP	on ASEs		on CSEs	
Heterozygosity^1)^	0.24	(S.E. 0.03)	0.19	(S.E. 0.03)
SNP density^2)^	0.030%		0.037%	
#HITs accordance with GT-AG rule^3)^	35/47	(74%)	100/175	(57%)
Splice score with matched allele^4)^	8.534	(S.E. 0.454)	7.835	(S.E. 0.600)
Splice score Δ two alleles^5)^	8.225	(S.E. 0.087)	8.235	(S.E. 0.128)

1) Average heterozygosity of sdSNPs.

2) Density of SNPs in splice dinucleotides. Note that the SNP density in intron regions is 0.139%.

3) Number of transcripts whose splice sites satisfy the GT-AG rule with the predicted allele/number of transcripts performed allele prediction assuming LD with the nearest cSNP. See Process 5 in Figure 2.

4) Average score of splice sites with the predicted alleles meeting the GT-AG rule. Splice scores were calculated using the MaxEnt program.

5) Average differences in score between the two splice sites from the two alleles of each sdSNP.

### Conservation of exons flanked by SNPs

**Figure 3:**
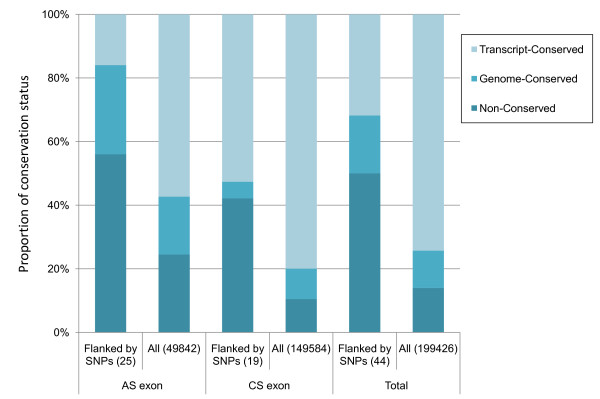
**Graph showing proportions of conservation statuses of exons in human and mouse genome**. Human exons were categorized according to conservation status of mouse homologous exons. A non-conserved exon is a human exon that does not align with a mouse genomic region using a threshold of 70% for coverage and 60% for identity. A genome-conserved exon is a human exon whose alignment with a mouse genomic region exceeds the above thresholds. A transcript-conserved exon is a human exon whose alignment with a mouse transcript exceeds the threshold [51]. Numbers shown at "Flanked by SNPs" and "All" in each category on the horizontal axis are numbers of genomic regions of sdSNPs (Process 7 in Figure 2) and of all exons in the human genome (Panel A of Table 1 in [51]).

We examined the conservation statuses of exons flanked by sdSNPs by aligning genome sequences with full-length transcripts of human and mouse. The conservation statuses of exons flanked by sdSNPs are available for 44 sdSNPs (i.e., 25 out of 53 sdSNPs at AE dinucleotides and 19 out of 159 sdSNPs at CE dinucleotides; Process 6 in Figure 2). These exons flanked by sdSNPs were classified into three categories according to whether exon and genome sequences are conserved between mouse and human, i.e., transcript-conserved, genome-conserved, and non-conserved (see Methods). The proportions of these three categories were compared between exons flanked by sdSNPs ("Flanked by SNPs" in Figure 3) and all exons in the human ("All" in Figure 3). The proportion of non-conserved exons was dramatically increased when exons were flanked by sdSNPs (Flanked by SNPs (50%) vs. All (14%), χ^2 ^= 47.47, *P *< 10^-12^, Total in Figure 3). This tendency was also observed for both exon types when they were classified into ASEs and CSEs.

### Allele prediction and matching to the GT-AG rule

To predict the alleles of sdSNPs for each transcript, we searched another SNP located in the transcribed region (cSNP) and combined both alleles of these two SNPs, supposing that linkage disequilibrium (LD) held between them and that the combination of alleles observed in the BAC sequences used for the reference genome was a haplotype (see Methods). Before applying this prediction to sdSNPs, we predicted alleles of all transcripts in H-InvDB and found that 98.8% of 3 197 363 CE dinucleotides and 95.7% of 177 136 AE dinucleotides followed the GT-AG rule. We could predict alleles in 47 out of 67 transcripts with sdSNPs at AE dinucleotides and in 175 out of 529 transcripts with sdSNPs at CE dinucleotides (Process 4 in Figure 2). Within these, we observed that the predicted alleles of the SNPs satisfied the GT-AG rule in 35 (74%) and 100 (57%) transcripts with sdSNPs at AE and CE dinucleotides, respectively (Process 5 in Figure 2). In this process, we defined sdSNPs as "constantly consistent" when only one allele of sdSNP was predicted to be consistent with the GT-AG rule in every transcript. sdSNPs whose predicted allele was inconsistent with the rule in every transcript were defined as "constantly inconsistent." When both alleles of a sdSNP were predicted (i.e., one allele was predicted to be consistent with the rule in some transcripts, but the other allele of the identical SNP was predicted to be inconsistent in other transcripts), the sdSNP was defined as "both cases." The six sdSNPs at CE dinucleotides were predicted to be "both cases;", accordingly, these six sdSNPs were counted in both categories--consistent and inconsistent with the GT-AG rule. No "both cases" were predicted in sdSNPs at AE dinucleotides (Process 5 in Figure 2). Consequently, 46 sdSNPs at CE dinucleotides were placed in three categories according to whether their predicted alleles satisfied the GT-AG rule; there were 6 sdSNPs with both alleles, 27 sdSNPs with constantly consistent alleles, and 13 sdSNPs with constantly inconsistent alleles (Process 5 in Figure 2). Among sdSNPs in AE dinucleotides, 24 and 12 were predicted to have constantly consistent alleles and constantly inconsistent alleles, respectively (Process 5 in Figure 2). Dinucleotide patterns obtained by the prediction of the SNP alleles are presented in Table 2. In AE dinucleotides, the most abundant non-canonical dinucleotide pattern was GT-AC, which was found in three transcript-SNP pairs involved in three SNPs (Table 2). In CE dinucleotides, the most abundant non-canonical dinucleotide patterns were GT-TG and GT-AA, which were found in 37 transcript-SNP pairs and 9 SNPs, respectively (Table 2). The widely known non-canonical splice dinucleotide GC-AG was observed in one out of 12 (8%) and seven out of 75 (9%) transcript-SNP pairs not satisfying the GT-AG rule at AE and CE dinucleotides, respectively.

**Table 2:** Number of transcript-SNP pairs (number of SNPs)

		Constituitive			Altenative
		
		Exclusive case^1)^	Shared case^2)^	Total case	Exclusive case^1)^
**Canonical**		87	13	100	35
	GT-AG	(27)	(6)	(33)	(24)

**Non-canonical**	GC-AG	0	7	7	1
		(0)	(3)	(3)	(1)
	AT-AG	1	2	3	3
		(1)	(2)	(3)	(3)
	CT-AG	6	0	6	1
		(3)	(0)	(3)	(1)
	GA-AG	0	0	0	1
		(0)	(0)	(0)	(1)
	GT-AA	9	0	9	1
		(4)	(0)	(4)	(1)
	GT-AC	5	0	5	3
		(1)	(0)	(1)	(3)
	GT-AT	0	1	1	1
		(0)	(1)	(1)	(1)
	GT-GG	6	0	6	0
		(2)	(0)	(2)	(0)
	GT-TG	37	0	37	1
		(1)	(0)	(1)	(1)
	TT-AG	1	0	1	0
		(1)	(0)	(1)	(0)
	Subtotal	65	10	75	12
		(13)	(6)	(19)	(12)

**Total**		152	23	175	47
		(40)	(12)	(52)	(36)

1) Only a single allele is predicted for all transcripts. This includes all SNPs flanked by AS exons.

2) Both alleles were predicted depending on transcript. This was found only in constitutive exons.

### Comparison of splice site scores

The observation that more predicted alleles followed the GT-AG rule at AE dinucleotides (35/47, 74%) than at CE dinucleotides (100/175, 57%) may suggest that recognition of CSE is robust against SNPs at their dinucleotides, while recognition of ASE is sensitive to SNPs (Table 1). We checked whether exon strength of ASEs was generally weaker than that of CSE, and whether this explains the observation.

**Figure 4:**
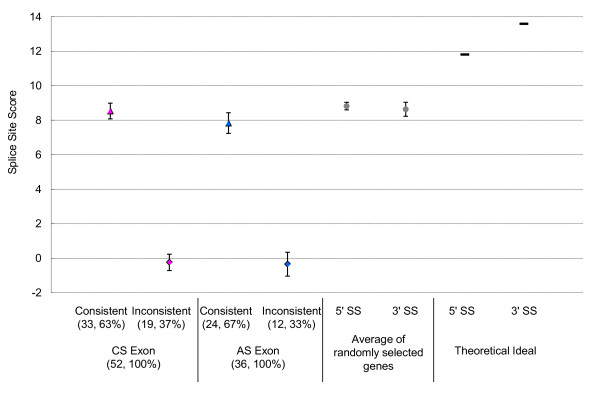
**Splice site scores shown by exon type and consistency between allele prediction and the GT-AG rule**. The average splice site scores for four categories, defined by whether predicted alleles were consistent with the GT-AG rule (consistent; triangle) or not (inconsistent; diamond), and whether they were located at a GT-AG site adjacent to CSE (pink symbol) or to ASE (blue symbol). Numbers in parentheses are numbers of cases and percentages. The six sdSNPs in CE dinucleotides are included in both consistent and inconsistent categories. Averages of randomly selected genes were calculated using values for *ARG2 *and *CFTR *genes (gray symbol) in Table 2 of reference [59]. These splice scores were calculated using MaxEntScan [56,57], and theoretically ideal scores are given in reference [59].

Figure 4 indicates that the average scores of splice site sequences including SNP alleles consistent (triangle) and inconsistent (diamond) with the GT-AG rule were 7.835 and -0.348 in ASEs and 8.534 and -0.243 in CSEs. Consequently, SNPs altered splice site scores to an average of 8.225 and 8.235at AE and CE dinucleotides, respectively (Figure 4, Table 1). This does not necessarily suggest that ASEs are weak exons. In addition, it does not imply that SNPs at AE dinucleotides change more drastically with exon strength than those at CE dinucleotides (Figure 4).

**Figure 5:**
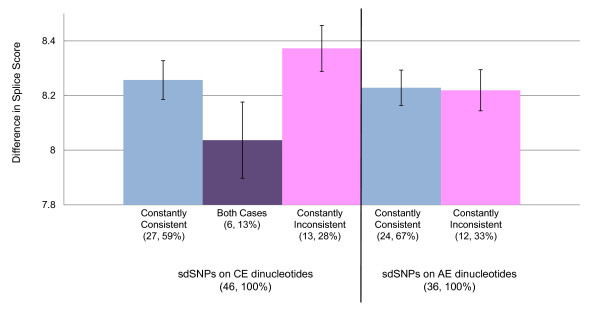
**Difference in splice site score between two alleles**. sdSNPs were categorized according to flanking exon types and SNP allele prediction. When only one allele of a sdSNP was consistent with the GT-AG rule in every transcript, the sdSNPs was defined as "constantly consistent" (blue), and those that were inconsistent with the rule in every transcript were defined as "constantly inconsistent" (pink). When both alleles of a sdSNP were predicted (i.e., one allele was predicted to be consistent with the GT-AG rule in some transcripts, but the other allele of the identical SNP was predicted to be inconsistent in the other transcript), the sdSNP was defined as "both cases" (purple). The six sdSNPs at CE dinucleotides were predicted to be "both cases," while no "both cases" was predicted for sdSNPs at AE dinucleotides (Process 5 in Figure 2). Numbers in parentheses are numbers of cases and percentages. Average differences of the score in sdSNPs at AE and CE dinucleotides are depicted right and left of the thick vertical bar, respectively.

The differences in splice site scores between two alleles for each SNP are shown in Figure 5. The sdSNPs for which both alleles were predicted showed slightly lower differences than others, although differences between other categories were not significant.

We did not find any evidence to suggest that multiple sdSNPs interact with each other to maintain exon strength. First, exons flanked by multiple sdSNPs and introns with a sdSNP at both ends were not observed. Second, a splice dinucleotide located in two consecutive sdSNPs (rs3667 and rs1130151) was observed; however, these did not affect our analysis because the predicted haplotype did not follow the GT-AG rule, i.e., the AA haplotype was predicted at the 3' splice site.

## Discussion

The SNP dataset includes validated SNPs only (see Methods). The heterozygosities of these selected SNPs indicate that most of them are common SNPs (Additional file 1, Tables S1, Additional file 1, Table S2); the average heterozygosities of sdSNPs in AE and CE dinucleotides were 0.24 (S.E. 0.03) and 0.19 (S.E. 0.03), respectively (Table 1); the difference was not significant (Aspin-Welch's *t *= 1.15, df = 60, *P *> 0.05). The SNP validation and heterozygosity data therefore suggest that SNPs observed in sdSNPs are not mutations related to serious disease but polymorphisms that are maintained in the human population.

All comparisons of various properties of the sdSNPs, including average heterozygosity, SNP density, the ratio of predicted alleles satisfying the GT-AG rule, and splice scores using the predicted allele, show no significant differences between ASEs and CSEs (Table 1). This suggests that sdSNPs are maintained regardless of whether the flanking exon is of the ASE or CSE type.

We mapped 212 sdSNPs in the human genome by searching the total human transcriptome. It is worth noting that 159 (75%) of these sdSNPs mapped to CE dinucleotides, and 53 sdSNPs (25%) mapped to AE dinucleotides (Process 3 in Figure 2). The 53 sdSNPs mapped to AE dinucleotides suggests that these sdSNPs are potential candidates for AS polymorphisms, which are SNPs that alter the splicing pattern [37]. In contrast to sdSNPs at AE dinucleotides, it is an intriguing discovery that sdSNPs exist at CE dinucleotides: this means that exons adjacent to these SNPs would be recognized constitutively regardless of allele. One explanation for the existence of sdSNPs at CE dinucleotides is that they represent an inconsistency between the definition of CSE based on publicly available human transcript data and on diversity in exon usage, which is affected by cell or tissue type, developmental stage, external stimuli [38,39], and genetic variation [40-44]. The number of ASEs is increasing as detection methods develop [29,45,46]. Because we defined CSEs based on publicly available human transcript data, our CSE category may include some ASEs if defined according to deep sequencing technology. Moreover, the number of specimens used for the current full-length cDNA data is smaller than that for SNP surveys in the dbSNP database. This may also lead to a difference between our definition of ASEs and that after taking polymorphism into consideration. Thus, CSEs are defined arbitrarily and vary with the amount of transcript data, which means that the ASEs in our dataset are likely to be highly alternatively spliced but that the CSEs are not necessarily constitutive. Comparison between genome and transcript sequences using common numerous specimens may resolve this uncertainty.

Another explanation is that CSE is robust against SNPs at their splice dinucleotides because of an unknown mechanism that can work even if the GT-AG rule is partially violated. U1-independent splicing may be an example that supports this explanation [47,48]. Although the detailed mechanism of U1-independent splicing is unclear and further research is needed, hF1γ intron 9, whose 5' splice site sequences are different from the consensus sequence at positions -3 and +5, spliced out without U1 snRNP. In this way, the 5' splice sites of these CSEs may be relaxed from selective pressure, which leads to maintenance of the sdSNPs at CE dinucleotides.

Our observation that the proportion of non-conserved exons was higher than that of conserved exons when the exons were flanked by sdSNPs suggests a relationship between exons with sdSNPs and newly generated exons (Figure 3). A question arises whether maintained sdSNPs lead to the generation of new exons, or whether newly generated exons bring about the maintenance of new flanking sdSNPs. Previous studies reported a lower degree of conservation of ASEs than CSEs and constructed models in which exons were generated as ASEs [21,49,50]. A previous study using the same datasets as the present study also showed that a limited proportion (6%) of AS variants was conserved between human and mouse [51]. Taking account of these, it is unlikely that as many CSEs are generated as ASEs. Consequently, our finding of no significant difference between ASEs and CSEs regarding sdSNPs suggests only a low possibility that sdSNPs lead to the generation of new exons. Instead, newly generated exons can be considered to serve additional functions and to be subject to relaxed selective pressure, in accord with the relaxation that occurs with duplicated genes (i.e., evolution by gene duplication) [52]. Our results suggest that newly generated exons allow the maintenance of flanking sdSNPs because of relaxed selective pressure on these exons.

## Conclusions

We found 212 validated sdSNPs in the dbSNP database that were consistent with the GT-AG rule at either allele. Proportion of non-conserved exon was higher when the exons were flanked by sdSNPs. We found no significant difference in the properties of sdSNPs between the two types of flanking exon (ASEs and CSEs). This includes average heterozygosity, SNP density, the proportion of predicted alleles satisfying the GT-AG rule, and scores for splice sites formed with predicted alleles.

The sdSNPs flanking ASEs can be explained by SNPs that alter the splicing pattern by their alleles, while the existence of sdSNPs flanking CSEs requires an explanation for constitutive splicing even when an expressed allele violates the GT-AG rule. Although this may be explained by the arbitrariness of the definition of the CSE, which varies among dataset, it suggests that a novel splicing mechanism makes CSEs robust against sdSNPs, such as U1-independent splicing.

Taking account of previous studies suggesting an exon generation model through ASEs, there is little likelihood that sdSNPs provide as much opportunities to generate CSEs as ASEs. Our finding of similar properties of sdSNPs between ASEs and CSEs does not suggest the possibility that sdSNPs are exon generators. Instead, the increased proportion of non-conserved exons found in exons flanked by sdSNPs suggests the hypothesis that sdSNPs are maintained at the splice dinucleotides of newly generated exons whose negative selection pressures are relaxed.

## Methods

### Determination of exon/intron structure using H-InvDB

We used a comprehensive annotation resource for all human transcripts released by the H-Invitational Database 4.6 (H-InvDB_4.6 [30]), which includes gene structure, transcript mapping location, and splice isoforms [31]. These data from H-InvDB_4.6 include all of the publicly available 120 558 human mRNAs mapped onto the human genome sequence (NCBI build 36.1) [53], forming 34 699 gene clusters, each of which we regarded as a putative "locus" (Figure 1A).

The H-Invitational consortium discriminates ASEs from CSEs using the H-Inv transcripts as previously described [32,33]. The ASEs obtained include the following types: cassette, internal acceptor, internal donor, mutually excusive, and retained intron. H-Inv transcripts with the same gene structure were grouped, and a representative AS variant (RASV) was selected from each group [32,54].

From all H-Inv transcripts, 80 997 were identified as AS variants and 41 289 were selected as RASVs [33]; these contained 107 606 ASEs and 298 102 CSEs (a total of 405 708 exons). We determined splice dinucleotides of all exons included in the filtered H-Inv transcripts using the genome position annotated in H-InvDB_4.6 (Figure 1A). We calculated the number of splice sites in the human genome by mapping all the RASV exons into the human genome.

### Extraction of SNPs at splice dinucleotides from dbSNP build 127

We searched for SNPs located at splice dinucleotides using the downloaded data of dbSNP build 127 [55]. The downloaded SNP data were mapped onto transcript positions (Figure 1A). We classified these retrieved SNPs located at splice dinucleotides into the following two groups according to the determination of ASEs and CSEs by H-DBAS: splice sites adjacent to ASEs; and those of CSEs, [32,33]. We selected validated SNPs from dbSNP to avoid inserting sequencing errors into our dataset. Such errors are prone to occur at the G base of 3' splice dinucleotides because of suppression of G after incorporation of A [18]. We removed the SNP-transcript pairs in which neither allele followed the GT-AG rule (Process 3 in Figure 2).

### Calculation of SNP density

To calculate SNP densities in splice dinucleotides of ASEs and CSEs, we obtained the total length of each splice dinucleotide region in the genome by mapping splice dinucleotides of all RASVs given by H-DBAS [32,33] onto the human reference genome sequence (NCBI build 36.1) [53]. For the comparison with average SNP density in the intron region, we defined the total intron length as the region obtained by subtracting the total length of exons from all representative transcripts in H-InvDB_4.6. We excluded the region when an intron overlapped with an exon of another representative transcript. Thus, we counted validated SNPs within the total intron region and calculated the total length of the intron.

### Allele prediction of SNPs at splice dinucleotides and inspection of agreement with the GT-AG rule

Since splice dinucleotides occur at both ends of introns, transcript sequences do not contain splice dinucleotides. To predict the SNP allele at splice dinucleotides for each transcript, we searched for another SNP that was most closely located at the exon neighboring the splice dinucleotides in every transcript. We extracted alleles of these two SNPs from published BAC clone sequences used for the human genome project [53]. We presumed the combination of alleles of these two SNPs was a haplotype, assuming LD between them. We also presumed that the other combination of the other alleles at the two SNPs formed another haplotype. According to the allele of the exonic SNP on a transcript, we determined which haplotype corresponded to each of the transcripts (Figure 1B). In the case of Figure 1B, transcript 1 was estimated as the same haplotype as the BAC sequence because the sequence at the SNP1 position on the BAC sequence is A, and transcript 1 also has A at the same position. Consequently, because the SNP2 position of the BAC sequence has G, the SNP2 allele of the transcript 1 was estimated as G. Transcript 2 was estimated as a different haplotype from the BAC sequence because of the difference in sequence at the SNP1 position, which led to the prediction that the SNP2 allele is different from the haplotype of the BAC sequence. In this case there are four possible combinations of the two alleles (table in Figure 1B). Among these, however, two combinations (A at SNP1 and G at SNP2, T at SNP1 and C at SNP2) are expected to be haplotypes, assuming LD between the two SNPs.

For all transcripts, we examined whether the allele of each sdSNP predicted from the haplotype of the transcript agreed with the expectation from the GT-AG rule.

### Maximum entropy scores

To estimate the strengths of 5' and 3' splice junctions in exons adjacent to SNPs at their splice dinucleotides, we calculated their splice site scores using the splice site models of MaxEntScan introduced by Yeo and Burge [56]. MaxEntScan is based on an approach to modeling short sequences that takes into account adjacent and nonadjacent dependencies using large datasets of human splice sites, based on the principle of maximum entropy. These splice site models assign a log-odds ratio as a MaXEnt Score to 9-mer (5' splice site) and 23-mer (3' splice site) sequences. The higher the assigned score, the higher the probability that the sequence is a true splice site. We downloaded the Perl scripts of MaxEntScan via the Internet [57] and applied this method to both splice junction sequences taken from BAC clones used for the human reference genome and replaced SNP sites with another allele.

### Inspection of exon mapping

Given two alleles of a SNP on a splice dinucleotide, in some cases there are two possible exon/intron boundaries that agree with the GT-AG rule (Figure 1C). One of these is the original boundary defined by H-InvDB ("Boundary-1 Allele-G" in Figure 1C), while the other is shifted one base pair ("Boundary-2 Allele-A" in Figure 1C). In this case, we selected the boundary that included the highest splice site score between two alleles' scores. We eliminated SNP-transcript pairs scoring negative values for both alleles when we summarized splice scores and compared them between ASEs and CSEs (Process 4 in Figure 2).

### Classification of sdSNPs by conservation status between human and mouse genomes

For the sdSNPs that were classified according to the type of flanking exons as ASEs or CSEs (Process 2 in Figure 2), filtering out those for which both alleles did not satisfy the GT-AG rule (Process 3 in Figure 2), we subclassified according to the conservation status of flanking exons between human and mouse genomes (Processes 6 and 7 in Figure 2). We used exon classification as performed in a previous study [51] and published through H-DBAS [32,33]; these references contain a detailed description of the method. Briefly, the criterion of the classification is as follows [58], human and mouse genome sequences that mapped with exons of their transcripts were aligned. If a human exon was conserved with respect to that of the mouse counterpart exon in the genome alignment with coverage and identity greater or equal in the threshold of 70% and 60%, respectively, it was defined as transcript-conserved. If a human exonic region aligned to the mouse genomic region exceeded these thresholds where no counterpart mouse exon was mapped, it was defined as genome-conserved. If a human exonic region in the genome did not align with the mouse genome using the thresholds stated above, the exon was defined as non-conserved. Because this conservation status was defined by genome position, the conservation status of a given sdSNP was determined uniquely even if multiple transcripts included the exon flanking the sdSNP (Processes 6 and 7 in Figure 2).

## Authors' contributions

MKS designed the study, performed the statistical analysis, and drafted the manuscript. YH scripted the in-house pipeline. JT analyzed AS variants and performed the evolutionary analysis of exon conservation. TG participated in the design and execution of the study. TI conceived the study and performed interpretation of the data. All authors read and approved the final manuscript.

## Supplementary Material

Additional file 1 **Supplemental tables**. Zip file containing the supplemental tables and their legends. Click here for file
