# Supplementary material for: A comprehensive survey of human polymorphisms at conserved splice dinucleotides and its evolutionary relationship with alternative splicing
Source: BMC Evol Biol. 2010 Apr 30;10:122. doi: 10.1186/1471-2148-10-122 (PMC2882926; doi:10.1186/1471-2148-10-122)
Supplement: Additional file 1 — Supplemental tables. Zip file containing the supplemental tables and their legends. [file 1471-2148-10-122-S1.ZIP › AdditionalFiles/LegendAdTables.rtf]

Legends of Additional Tables
<<Title>>
Table S1: SNPs located on splice dinucleotides (sdSNPs) in human transcriptome
Table S2: Summary of selected human transcript and SNP pairs

<<File Names>>
Table S1:
TableS1_altY.txt - list of transcript and ASE-SNP pairs whose estimated allele is consistent with GT-AG rule
TableS1_altN.txt - list of transcript and ASE-SNP pairs whose estimated allele is not consistent with GT-AG rule
TableS1_conY.txt - list of transcript and CSE-SNP pairs whose estimated allele is consistent with GT-AG rule
TableS1_conN.txt - list of transcript and CSE-SNP pairs whose estimated allele is not consistent with GT-AG rule

Table S2:
TableS2_a.txt - Summary of transcript and ASE-SNPs pair
TableS2_b.txt - Summary of transcript and CSE-SNPs pair

<<Legends>>
Table S1:

Column Name of Table S1

#1 rsSNP_ID: rs SNP ID in dbSNP
#2 allele
#3 genome coordinate	
#4 SNP strand
#5 heterozygosity
#6 number of locations SNP mapped: 'OneMap' or 'MultiMap', see List of Abbreviation 
#7 validation of SNP performed by dbSNP
#8 HIT ID. + intron No.
#9 intron start
#10 intron end
#11 intron strand
#12 splice site no*
#13 splice site base**
#14 matching GT-AG rule
#15 chromosome no.

*splice site no.

         12   34
genome XXGTxxxAGXX
         ||   ||  
cDNA   XXGT...AGXX

**splice site base: When two consecutive sdSNPs occupy a splice dinucleotide, the predicted haplotype is denoted.

Table S2:

Column Name of Table S2

#1 HIT ID: H-InvDB Transcript ID
#2 Intron No: _th intron in the transcript
#3 SitePos: see Figure 1a
#4 GeneSybl: HUGO gene symbol
#5 rs ID: rs SNP ID in dbSNP
#6 rsAlleles: alleles in dbSNP
#7 Ancestral Allele: ancestral allele estimated by NCBI and shown in dbSNP
#8 Ori/Str(rs): orientation and strand of the SNP, see ftp://ftp.ncbi.nih.gov/snp/database/Illumina_top_bot_strand.note.txt
#9 number of locations SNP mapped: 'Y'='OneMap' or 'N'='MultiMap', see List of Abbreviation
#10 Het: heterozygosity with standard error shown in dbSNP
#11 AncAlvsGT-AG: whether estimated ancestral allele is consistent with GT-AG rule

<<List of Abbreviation>>
sdSNP: SNP located on splice dinucleotide
ASE-SNP: sdDNP flanking alternatively spliced exon
CSE-SNP: sdDNP flanking constitutively spliced exon
HIT: H-InvDB transcript
OneMap, MultiMap: 'OneMap'- SNP mapped on one location in genome, 'MultiMap'- mapped more than two locations, which is defined in dbSNP, see http://www.ncbi.nlm.nih.gov/SNP/snp_db_table_description.cgi?t=SNPChrPosOnRef
